# A Neural Network and Optimization Based Lung Cancer Detection System in CT Images

**DOI:** 10.3389/fpubh.2022.769692

**Published:** 2022-06-07

**Authors:** Chapala Venkatesh, Kadiyala Ramana, Siva Yamini Lakkisetty, Shahab S. Band, Shweta Agarwal, Amir Mosavi

**Affiliations:** ^1^Department of ECE, Annamacharya Institute of Technology and Sciences, Rajampet, India; ^2^Department of IT, Chaitanya Bharathi Institute of Technology, Hyderabad, India; ^3^Future Technology Research Center, College of Future, National Yunlin University of Science and Technology, Douliou, Taiwan; ^4^SAGE University, Indore, India; ^5^John von Neumann Faculty of Informatics, Obuda University, Budapest, Hungary; ^6^Faculty of Civil Engineering, TU-Dresden, Dresden, Germany; ^7^Institute of Information Engineering, Automation and Mathematics, Slovak University of Technology in Bratislava, Bratislava, Slovakia

**Keywords:** cancer, lung cancer, machine learning, artificial intelligence, deep learning, cancer detection

## Abstract

One of the most common causes of death from cancer for both women and men is lung cancer. Lung nodules are critical for the screening of cancer and early recognition permits treatment and enhances the rate of rehabilitation in patients. Although a lot of work is being done in this area, an increase in accuracy is still required to swell patient persistence rate. However, traditional systems do not segment cancer cells of different forms accurately and no system attained greater reliability. An effective screening procedure is proposed in this work to not only identify lung cancer lesions rapidly but to increase accuracy. In this procedure, Otsu thresholding segmentation is utilized to accomplish perfect isolation of the selected area, and the cuckoo search algorithm is utilized to define the best characteristics for partitioning cancer nodules. By using a local binary pattern, the relevant features of the lesion are retrieved. The CNN classifier is designed to spot whether a lung lesion is malicious or non-malicious based on the retrieved features. The proposed framework achieves an accuracy of 96.97% percent. The recommended study reveals that accuracy is improved, and the results are compiled using Particle swarm optimization and genetic algorithms.

## Introduction

The most well-known reason for death because of malignant growth is lung cancer. The second most habitually analyzed type of malignancy is lung cancer. Pneumonic nodules are apparent in the lung to evaluate metastases from different malignancies (1, 2). Computed tomography (CT) is the most significant image mode for assessing progress/crumbling and for observation and decision-making malignant lung growths. As a result of the precocious presentation of lung malignancy by CT, doctors can be suggested more productive treatments (3, 4). Guess and recuperating components for scattered sickness with precise malignancy stages are required for orderly and consoling treatment (5). The early conclusion of the period of lung malignancy is firmly connected to the patient's continuance rate (6). In clinical terms, the disease is known to be strange hyperplasia and significantly beyond what 200 sorts can influence the individuals (7). According to the ACS (American Cancer Society), lung malignancy is the main cause of death in both men and women in the United States. about a total of 2,28,820 new lung malignancy cases were estimated, with 1,35,720 deaths (8). It causes a larger number of deaths than other malignant tumors. Early recognition of tumorous lung nodules is the key factor for patient survival rate. When contrasted with chest X-ray imaging, CT perceives the tumorous nodules consistently at an underlying stage (9). Practically all radiologists use CT by exploring multiple pictures from a solitary patient. Thus, the exhaustion of the radiologists can prompt wrong analysis. Hence, the exact physical valuation measure is tedious and colossally inconsistent (10). A precise segment is noteworthy for the right valuation of nodule improvement and for the arrangement of malignant nodules (disease cells) from benign ones (non-disease cells). The reason for this work is to exactly recognize the nodules over the CT lung pictures.

The proposed instructional method pulls back, utlilizing a middle method to reduce confusion based on the CT image. Second, a crossover division approach is utilized to isolate the lung zone from its environmental factors. The proposed division strategy utilizes the Otsu thresholding to eliminate superfluous groups, consequently isolating the specific lung locales, and nodules of interest can be decisively characterized by cuckoo inquiry advancement. Third, the assortment of surface highlights for the particular nodule is undisturbed by parallel neighborhood examples at the feature stage. Finally, the highlights of the sectioned lung nodules are prepared by the CNN classifier to distinguish the lesions as malignant or non-malevolent.

## Related Work

In 2019, Ananya et al. (11) developed a multi-approach system for lung cancer categorization using genetics. They assessed false negatives and true positives for classification accuracy in this study, but not detected accuracy.

In 2019, Venkatesh et al. (12) developed an innovative approach to detect lesions based on a GA and LBP. This process achieves an accuracy of 90%.

In 2019. Preeti et al. (13) introduced a lung cancer detection framework based on the fuzzy c-mean clustering and SVM classifier techniques

In 2019, Senthil Kumar et al. (14) introduced an approach for detecting lung lesions using GCPSO. In this work, multiple optimization techniques are used to classify cancer in CT images. The process obtained a precision of 95%.

In 2018, Perumal et al. (15) proposed an ABC algorithm for malignancy recognition and classification. This guidance attained a 92% accuracy.

In 2017 Ammar et al. (16) established an early diagnostic architecture for genetically altered tumor detection. In this study, the authors achieved an accuracy rate of 84%.

In 2017 Kamil et al. (17) introduced a DWT-based lung lesion detection system. In this method by using subtraction and erosion techniques images are analyzed to remove the cancer region. This approach yielded an accuracy of 89%.

In 2016 Mukesh et al. (18) introduced a DWT-based method for assessing a high volume of tissues in chest X-ray images. Using this method, the authors were able to achieve an accuracy of 86%.

In 2014 Santos et al. (19) described an area development and Hessian matrix to identify minor respiratory lesions. The presented approach achieves a classification accuracy of 88.4%.

In 2014, Jinsa and Gunavathri (20) reported an ANN-based lesion categorization technique. They were able to classify with an accuracy of 93.3%.

The principal gap has indeed been extended due to the lack of research publications that demonstrate computations. Because of poor directionality, slower processing, greater calculation time, and complex computations, the methods suggested by the authors mentioned above are less effective in all cases. As a result, an interactive technique for identifying lung cancer in CT images is suggested in this article, which uses the otsu threshold-based Cuckoo search algorithm, Local Binary Pattern for image retrieval, and CNN for classification to conquer all of the shortfalls of the existing methods. By selecting the most cost-effective strategy, optimization algorithms tend to produce a solution for image processing processes.

## Motivation and Contribution

Lung cancer is confirmed by physicians after a thorough examination of CT scans, which requires a lot of time and is not always accurate. To create imagery as precise, operational, and efficient as possible, state-of-the-art optimization techniques and image processing approaches were required. The proposed technology will aid doctors in accurately identifying lung nodules at an early stage, as well as studying the internal anatomy. As a part of the contribution, some glitches related to lung cancer detection are discussed here. The region of interest is retrieved using Otsu thresholding and cuckoo search optimization, which is a novel approach to segmentation. This proposed partitioning approach requires only a few parameters to precisely separate nodules of varied sizes and shapes.

### Proposed Methodology

**Figure 1:**
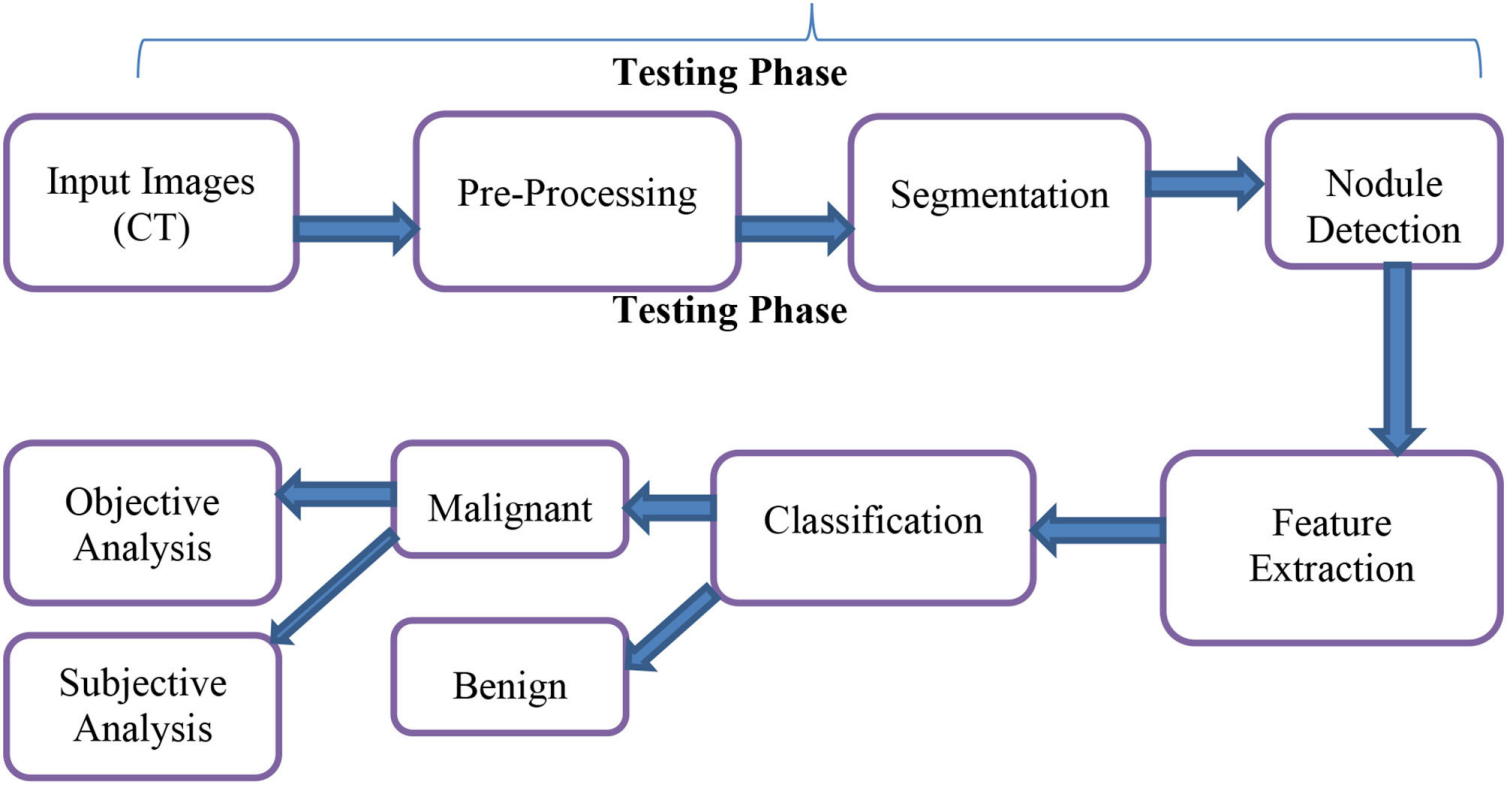
Architecture of proposed method.

Figure 1 depicts the prospective lung malignancy diagnostic procedure, which comprises five phases: (1) contrast enhancement and Noise reduction through pre-processing, (2) Otsu thresholding based cuckoo search algorithm to segment the lesion from its backgrounds, (3) retrieval of regions of concern, (4) retrieval of descriptors from segmented lung lesions, and, in the last phase, (5) SVM has been used to assess if the lesion was abnormal or normal. The next sections provide detailed descriptions of the above-mentioned phases.

### Image Acquisition

It is the basic step before proceeding with other critical steps. It is a method for processing a digital image from a database (21). Numerous sorts of scanners, such as X-Ray, MRI, and CT, are used to obtain the images. The CT image was captured using a CT scanner. It is a type of scanning that creates cross-section scans for each pixel (22).

### Pre-processing

The equations should be inserted in editable format from the equation editor. It is then procedure to improve image details. The basic idea is to suppress noise, which corrects undesired distortions and enhances the associated attributes of the image for subsequent processing (23). Because all techniques are sensitive to noise, efficient images pre-processed allow for better segmentation and, as a result, better classification. The size of the pixel area could be used to classify pre-processing procedures. Image enhancement employs these techniques. Enhancement operations operate on the image pixels of the neighborhood and the corresponding values of the neighborhood. Contribute quality to the images by decreasing noise and distortion (24).


$$f\left(x\right)=a_{0}+\sum_{n=1}^{\infty }\left(a_{n}cos\frac{n\text{π}x}{L}+b_{n}sin\frac{n\text{π}x}{L}\right)$$

#### Median Filtering

Salt and pepper noise can be found on CT scans. The finest features are obscured by these impacts. By keeping the frontier of the image as fine as possible, this filtering lowers salt and pepper noise (12). This filter gathers information from a sample within a non-averaged window (25). The edges of the filter are better managed than those of other linear filters. The following equations are used to get the median value.


(1) $$M\left(g\right)=\sum_{k=1}^{n}\left|x_{k}-g\right|.$$

(2) $$g=median\left\{x_{1},x_{2},\ldots \ldots \ldots .x_{n}\right\}.$$

### Otsu Thresholding Segmentation With Optimization

#### Otsu Thresholding

The goal of this strategy is to scour specified like-classes of pixels in a picture for the closeness of neighboring pixels in order to generate a concentrated image object. Separating the background and sub-regions in medical imaging is tough (26). The Otsu segmentation algorithm works better to “recognize” or “smear” the context contents of the front objects. It is an adaptive threshold binarization procedure proposed by OTSU in 1979. This procedure uses the highest within-class variance between the context and the target based on the rule of threshold assortment (27). It segments the image into the forefront and the contextual based on the characteristics of gray level values. If the finest threshold is attained, the gap between the two regions is the highest. The Otsu algorithm, in general, utilizes the greatest within-class variance. The larger the variance value, the wider the difference between the two areas, since variance is a useful determinant of uniform gray distribution. If some areas are wrongly segmented into contextual or if some contextual is segmented into areas, then the gap is too small between the two areas. As a result, if the variance among groups is higher, the likelihood of incorrect classification is lowered, resulting in cohesive segmentation.

The following is the main principle of OTSU-based threshold segmentation:

Let us call the gray values *g* and the number of pixels *nx*. Then


(3) $$P=\sum_{x=0}^{L-1}n_{x}=n_{0}+n_{1}+n_{2}+\ldots +n_{L-1}\ldots$$

where g = 0, 1,..., L-1, and P indicate the number of pixels. Suppose C1 and C2 are the two kinds of pixels. C1 pixels have a range of [0,x] while C2 pixels have a range of [x+ 1,L1].


(4) $$\sigma_{Gv}^{2}=\sum_{g}^{L-1}{(g-m_{Gv})}^{2}.$$

(5) $$\sigma_{Bv}^{2}=P_{1}{\left(m_{1}-m_{Gv}\right)}^{2}+P_{2}{\left(m_{2}-m_{Gv}\right)}^{2}$$

The below-mentioned calculations are used to compute the mean intensities.


(6) $$m_{1}=\frac{1}{P}\sum_{g}^{x}g.P_{g}$$

(7) $$m_{2}=\frac{1}{P}\sum_{g=x+1}^{L-1}g.P_{g}$$

(8) $$m_{Gv}=\sum_{g=0}^{L-1}g.P_{g}$$

where m 1 and m 2 are the C1 and C2 pixel average intensities, and m Gv is the global mean intensity. Lastly, the ratio τ , which is provided below, is used to determine the ideal threshold.


(9) $$\tau =\frac{\sigma_{Bv}^{2}}{\sigma_{Gv}^{2}}$$

#### Cuckoo Search Optimization

The cuckoo generation function is projected using this approach, which reduces the implications. A large number of nests are accessible during the search procedure. The location of the cuckoo egg has been discovered as a novel solution (28). The steps in the search procedure are as follows. A cuckoo bird places one egg at a time in a randomly chosen nest. The parasite nests were static, and the number of eggs in the nests would increase until they reach their highest level. When the cuckoo's egg is spotted, the host bird seems to have the choice of chucking the egg away or scrapping the nest and forming a new one.

The Levy flight theory has improved the CS algorithm (29). This CS technique is used to calculate the appropriate threshold for eliminating the lung nodule.

The following analogy is incorporated into the proposed technique for optimum selection (Algorithm 1):

**Algorithm 1:** Cuckoo search algorithm.

Step1:	Initialization parameters: n, Pa, & M where n=number of host nests; pa : probability of discovery of alien, M: maximum number of iterations
Step2:	Generate initial n host, n_i^t^
Step3:	Evaluate f(n_i^t^)
Step4:	Generate a new solution $n_{i}^{t+1}=n_{i}^{t}+\alpha ⊕Lev^{{}^{\prime }}y\left(\gamma \;\right)$
Where the symbol ⊕ is entry-wise multiplication, α>0 indicates the step size, Levy(γ)= *g*^−γ^(1 < γ ≤ 3)	
Step5:	Evaluate *f*$\left(n_{i}^{t+\;1}\right)$
Step6:	Choose a nest *n*_*j*_randomly
Step7:	If $\left(n_{j}^{t})>(n_{j}^{t+1}\right)$)> $\left(n_{j}^{t+1}\right)$ then Replace $n_{j}^{t}\;with\;n_{j}^{t+\;1}$
Step8:	Confiscate a worse nest with Pa
Step9:	Construct new nest using Levy flights
Step10:	Retain the best solutions

The new solution, which depicts the subcategory of thresholds, is depicted by the egg of a cuckoo. This is also utilized for segmentation of the lung nodule. The grade of eggs for each host nest is either 0 or 1, which mimics the segmentation procedure's threshold partition. Pa is the probability that a cuckoo's egg will be discovered by the host bird. Pa has a predefined threshold. It demonstrates the principle of removing the least relevant threshold subgroups and, as a result, removing these threshold values from further analysis.

### Feature Extraction

The LBP operator was established to determine texture in the first place (30). By thresholding an image with the central pixel value and taking the result as a binary quantity, the operator applies a mark to each pixel (31). The picture of the Lung CT could be considered as a micro-pattern structure that the LBP operator can well portray. The steps for extracting the characteristics are outlined below.

- Divide the window looking at into cells.
- In a cell, collate each pixel with its neighbors.
- If the value of the center pixel is larger than the value of the neighbor pixel, assign “1”; otherwise, assign “0”.
- A binary number is created by comparing all of the pixels.
- Lastly, over the cell, compute the histogram. The LBP value can be calculated using the expression $LBP_{X,Y\;}^{U}$where U represents uniform pattern and X,Y indicate neighborhood.

(10) $$LBP_{X,Y=\sum_{0}^{m-1}s\left(P_{s}-P_{c}\;\right)2^{s}}$$

(11) $$s(t)=\{\begin{array}{ll} 1 & t\geq 0\\ 0 & t<0 \end{array}$$

P_c_ is the gray value of the center pixel, P s is the intensity value of the location pixels (m = 0, 1,......m-1), and m is the image element well within range R where R is higher than zero (R > 0), creating a regionally oriented neighborhood set. After identifying each pixel in a picture, a histogram is created to define the texture image (32, 33).

## Classification

**Figure 2:**
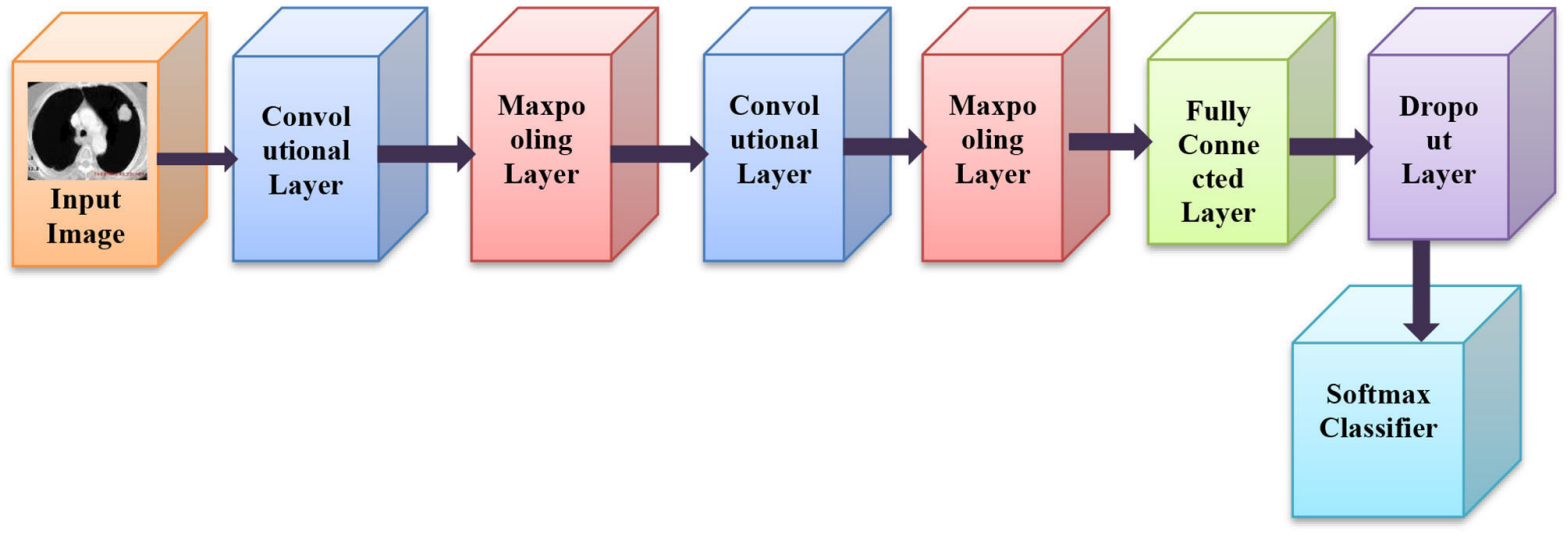
Architecture of CNN.

CNN belongs to DNN group which is comprised of numerous hidden layers, like RELU, fully linked, pooling and convolution layer, etc. CNN securities weights in the coevolutionary layer, which lowers the network latency, and enhances network performance (34). CNN's prominent features are prevalent weights, local networking, and neuronal 3D sizes. A feature map is created with a kernel by a convolution layer of diverse sub-regions of the input image (35). Then, a nonlinear function is added to the RELU layer to progress the convergence possessions when the error is small. The architecture of CNN is as shown in Figure 2.

In relation to complex layers, CNNs quite often incorporate pooling layers (36). They are principally used only for lessening the dimensions of the tensor and speeding up estimations (37). All such layers are simple. So, the image is to be split up into smaller portions in the pooling layer and for each portion, the maximum value is selected and then accomplished in some process for each portion (38). After being portioned, it is placed in the output in the respective position. RELU is a rectified linear unit, as well as a form of hidden layers. The activation function is most popularly used in neural networks, predominantly in CNNs (34).

## Simulation Results

Lung cancer is diagnosed in CT medical images using the novel cuckoo search algorithm and attributes are determined in this study. Lung cancer CT images were collected from a private hospital (Satyam diagnostic center, Anantapur). The adaptive threshold issue in this study is referred to as an optimization problem and it can be resolved using the CSA approach. In this study, the outcomes of the suggested method were compared to those of the PSO and GA algorithms. This work has been carried out by MATLAB software. When compared to open-source tools, MATLAB has a great affinity with deep learning techniques as well as hardware tools. Also, open-source tools have a hard time bringing all of the libraries together in one spot.

**Figure 3:**
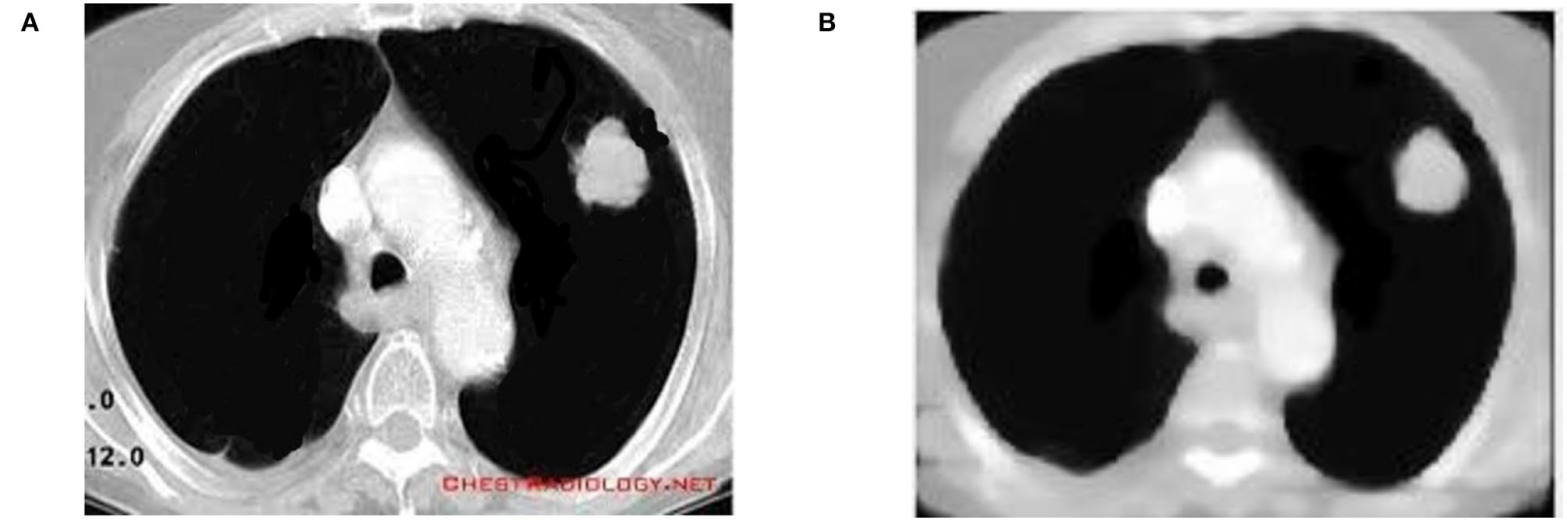
**(A)** Input CT image. **(B)** Filtered output.

This study relies on my prior work (39), in which the outcomes were produced using a Genetic algorithm and particle swarm optimization approaches in addition to LBP and CNN. Cuckoo search optimization is used in this work, together with CNN and LBP, to enhance the accuracy. The input and median filter output of CT lung cancer pictures are depicted in Figure 3. Low-frequency noise and distortion are common in CT scan images. To reduce noise and distortion, the input image is processed with a median filter.

**Figure 4:**
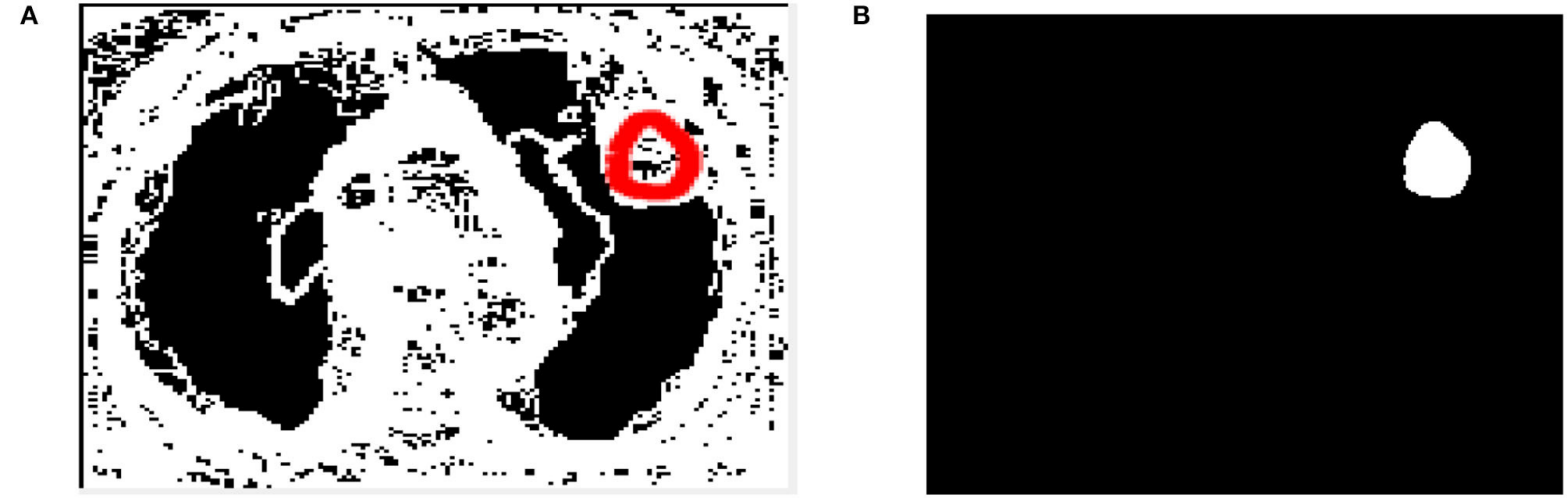
**(A)** Extracted output. **(B)** Segmented output.

To identify lung lesions in the CT image, it is split into multiple clusters and then optimized using the Otsu thresholding approach. The CT scan is first split using simple Otsu thresholding, which improves the segmented classes' variation, or “all class variance.” The result of the thresholding technique can be enhanced by processing it with cuckoo search optimization. Following partitioning, the image is subjected to LBP feature extraction, which extracts the textural features before extracting the detected output (see Figure 4).

**Figure 5:**
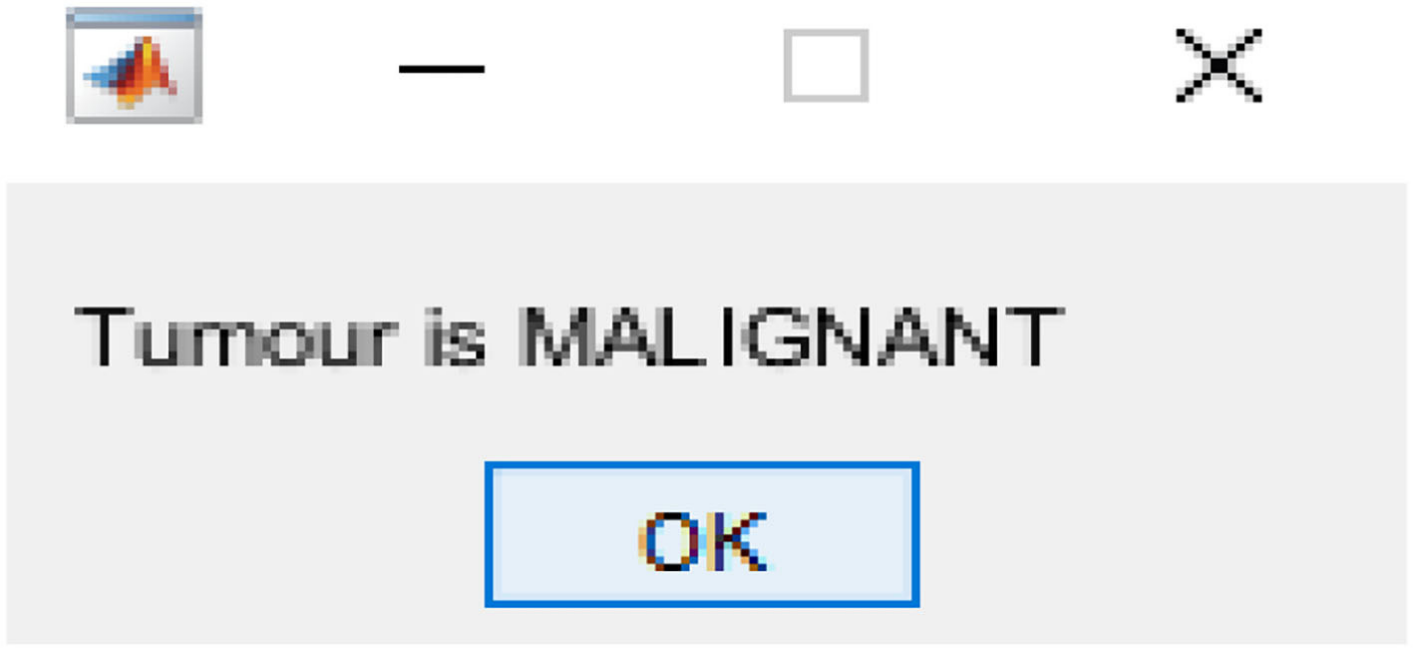
Classification output.

**Figure 6:**
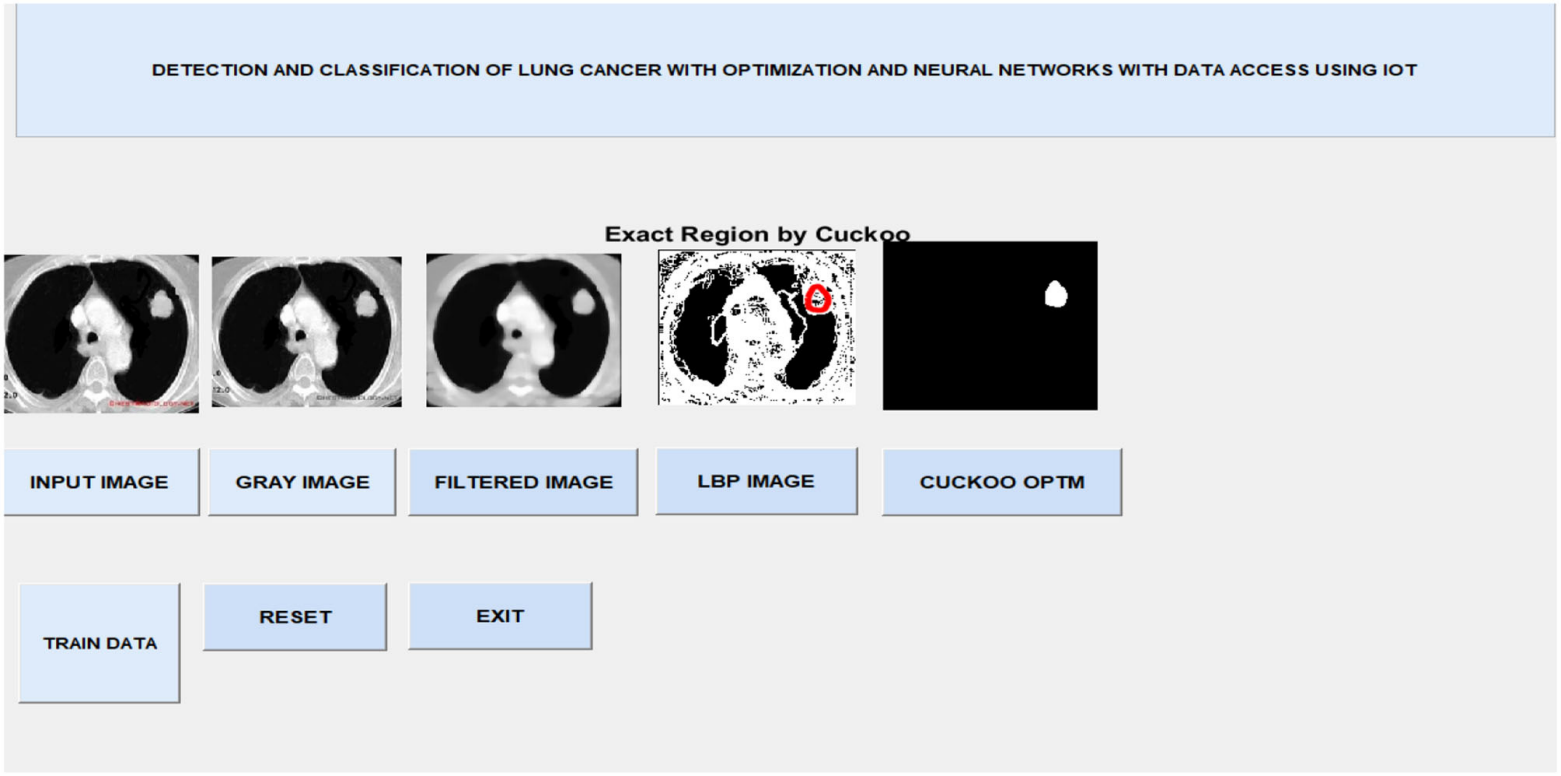
GUI output.

**Figure 7:**
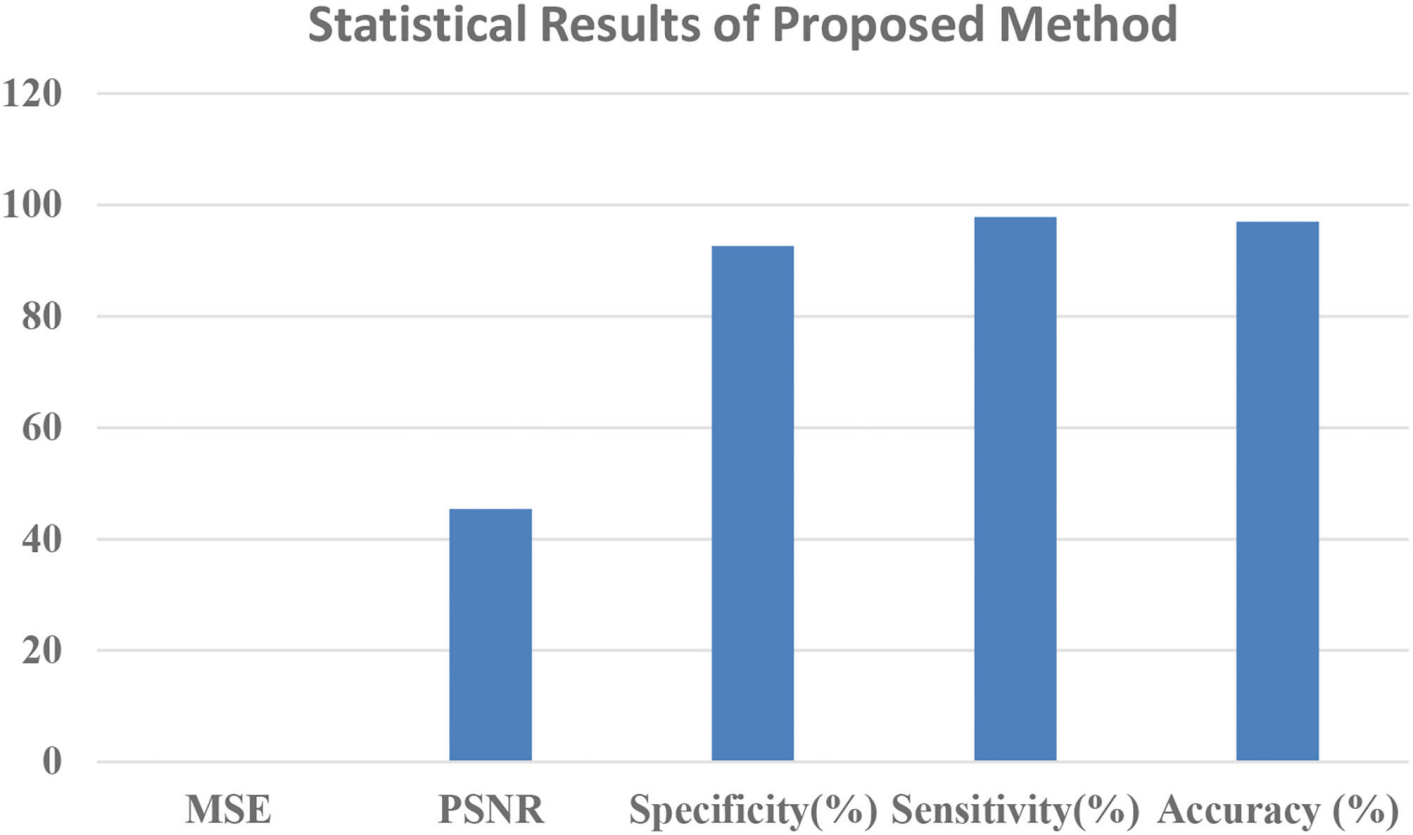
Statistical results graphical representation.

After image retrieval, the image is given to CNN classification, which assesses the image as normal or abnormal by showing a message such as “Tumor is MALIGNANT” or “Tumor is BENIGN,” as illustrated in Figure 5. The system's general function is created in a GUI, as seen in Figure 6. The statistical results like performance metrics obtained by 200 iterations for the input image are shown in graphical representation in Figure 7.

Table 1 showed that the suggested approach yields higher accuracy of 97%, the sensitivity of 97.8%, specificity of 92.6%, PSNR of 45.38%, and low MSE of 0.013 than conventional systems. These optimum results are obtained for 200 iterations (Our earlier proposed systems).

**Table 1:** Attributed obtained from the proposed method.

**Parameters**	**Proposed Method (CSO+CNN+LBP)**
MSE	0.013
PSNR (%)	45.38
Specificity (%)	92.672
Sensitivity (%)	97.806
Accuracy (%)	96.979

**Figure 8:**
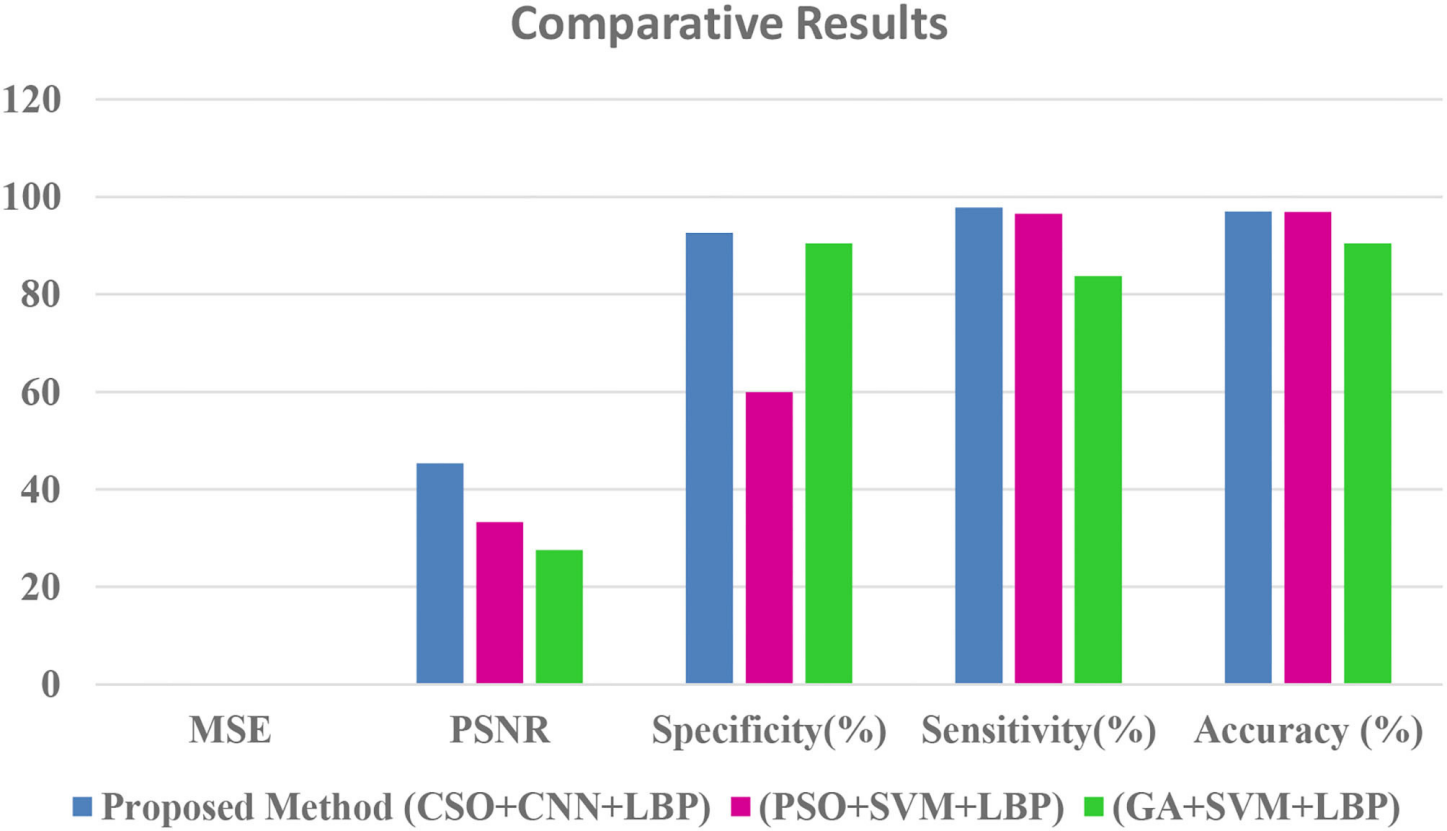
Comparative results graphical representation.

Table 2 shows that the suggested approach has higher accuracy (97%) than conventional systems (Our earlier proposed systems). The Comparative Results Graphical Representation is shown in Figure 8.

**Table 2:** Comparative Results with proposed method.

**Parameters**	**Proposed Method (CSO+CNN+LBP)**	**(PSO+SVM+LBP)**	**(GA+SVM**+**LBP)**
MSE	0.013	0.0301	0.0651
PSNR	45.38	33.2788	27.5311
Specificity (%)	92.672	60.0000	90.4950
Sensitivity (%)	97.806	96.5783	83.7143
Accuracy (%)	96.979	96.9391	90.4937

## Conclusion

In this article, a strong approach for recognizing lung cancer in CT images is developed. For exact cancer diagnosis in CT lung images, the Otsu thresholding-based cuckoo search optimization and CNN classifier approach were used. Based on the simulation findings, it is observed that the suggested method reliably segments CT images and detects lesions of various forms and sizes. Subsequently, the proposed approach comprises successive stages that continuously yield the last detection result. The techniques that are utilized in different stages are basic and simple to actualize.

Based on the simulation findings, the accuracy of the proposed framework' is calculated to be 96.97%, which is greater than any other demonstrative framework found in the literature. As for future work, a powerful strategy could be created by supplanting the CNN Classifier with a profound deep learning method and CAD tools. One may improve the structure for images of lung cancer in different modalities.

## Data Availability Statement

The raw data supporting the conclusions of this article will be made available by the authors, without undue reservation.

## Author Contributions

CV: conceptualization, methodology, software, and visualization. KR: data curation, writing—original draft, data analysis, and investigation. SL: software, validation, and editing. SB: software, validation, and editing. AM: supervision, writing—review & editing. SA: software, validation, and editing. All authors contributed to the article and approved the submitted version.

## Conflict of Interest

The authors declare that the research was conducted in the absence of any commercial or financial relationships that could be construed as a potential conflict of interest.

## Publisher's Note

All claims expressed in this article are solely those of the authors and do not necessarily represent those of their affiliated organizations, or those of the publisher, the editors and the reviewers. Any product that may be evaluated in this article, or claim that may be made by its manufacturer, is not guaranteed or endorsed by the publisher.
